# Supplementary material for: Century-old chromatin architecture revealed in formalin-fixed vertebrates
Source: Nat Commun. 2024 Jul 29;15:6378. doi: 10.1038/s41467-024-50668-4 (PMC11286846; doi:10.1038/s41467-024-50668-4)
Supplement: Supplementary file 1 — Supplementary Information [file 41467_2024_50668_MOESM1_ESM.pdf]

Supplementary Information

- **Supplementary Figures**

Century-old chromatin architecture revealed in formalin-fixed vertebrates

Erin E. Hahn[1], Jiri Stiller[2], Marina Alexander [1], Alicia Grealy [1], Jennifer M. Taylor [2], Nicola Jackson[3], Celine H. Frere[3] and Clare E. Holleley[1*]

[1]National Research Collections Australia, Commonwealth Scientific Industrial Research Organisation, Canberra, ACT 2601, Australia

[2]Agriculture and Food, Commonwealth Scientific Industrial Research Organisation, St Lucia, Queensland 4067, Australia

[3]School of the Environment, University of Queensland St Lucia, Queensland 4067, Australia

* Corresponding author: Clare Holleley E-mail: clare.holleley@csiro.au

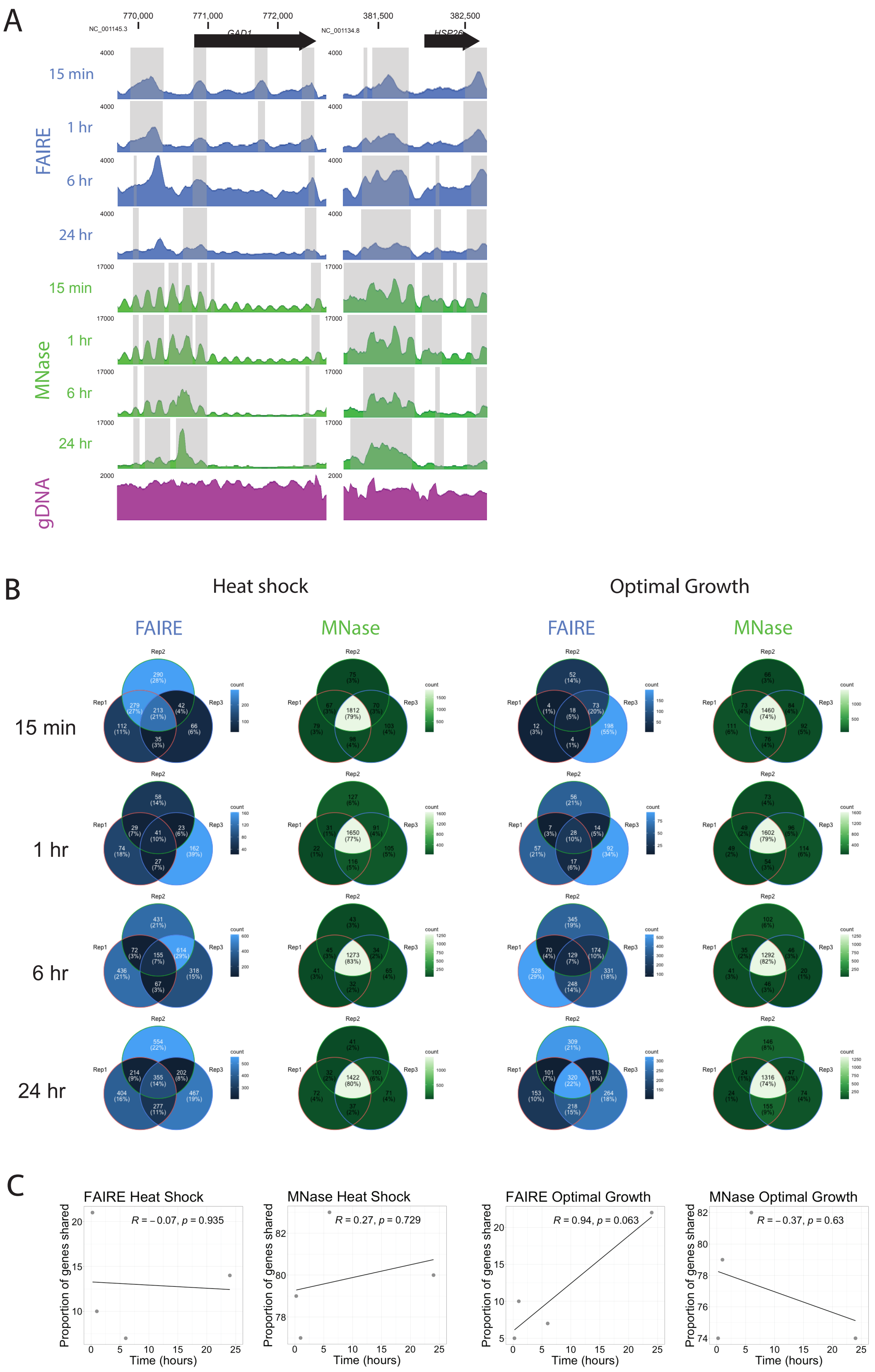

Supplementary Figure 1. Further Timepoints for Yeast alignments and Venn diagrams

A. Pooled occupancy values (FAIRE: blue, MNase:green) compared to gDNA extraction control (purple) with formaldehyde fixation for 15 min, 1 hr, 6 hr and 24 hr of heat shocked S. cerevisiae. Shading indicates regions with significant peak width shifts (FDR < 0.05) between treatment and input control. Upstream of highly upregulated GAD1 and HSP26 genes (log2FC = 3.8 and 9.02), changes in occupancy signal morphology are observed. The 5' FAIRE peak broadens, while the distinct 5' MNase nucleosome array transforms into a single peak. B. Venn diagrams demonstrate repeatability of the FAIRE (blue) and MNase (green) assays among technical replicates in yeast cultures grown under heat shock or optimal growth conditions fixed with formaldehyde. Numbers/proportions represent genes with significant peak gain (FDR < 0.05, log10Pval < -6) within 2 kb upstream of the TSS. Lighter colours indicate higher shared gene count. C. Correlation tests between proportion of genes shared between all three replicates in (B) and fixation time. Linear regression lines are fitted, and correlation coefficients (R) and p-value are provided for each time point.

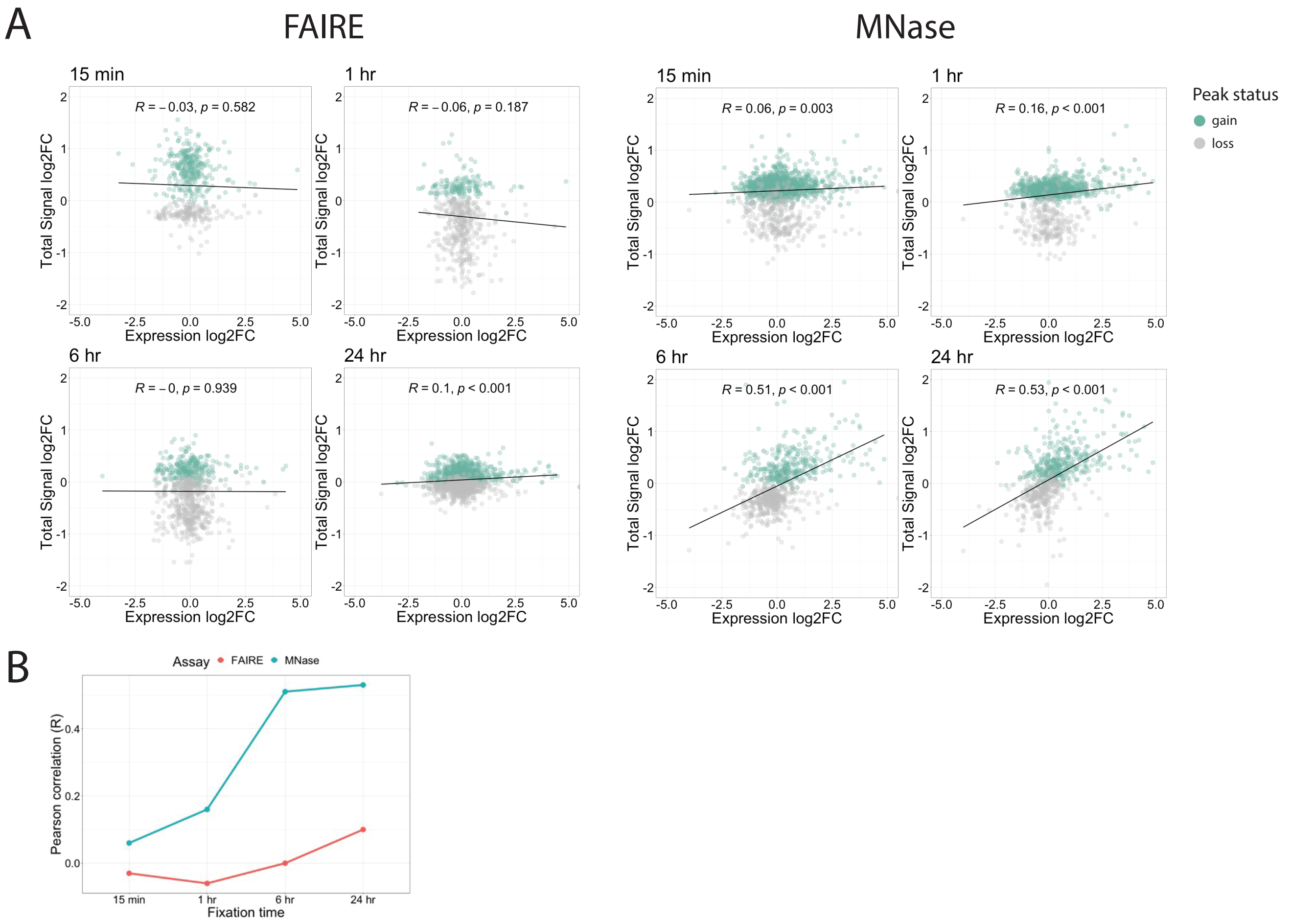

Supplementary Figure 2. Further Timepoints for yeast differential DANPOS & expression correlation

A. For the FAIRE and MNase assay of yeast fixed for between 15 min and 24 hr, total signal log2FC for genes with significant (FDR < 0.05) total peak signal change between pooled replicate heat shock and optimal growth conditions in the 2 kb region upstream of the TSS is plotted against expression log2FC measured by RNA-Seq. Genes are coloured green for signal gain or grey for signal or loss. Linear regression lines are fitted, and correlation coefficients (R) and p-value are provided for each time point. B. Summary plot of Pearson correlation (R) values for the MNase and FAIRE time series in A.

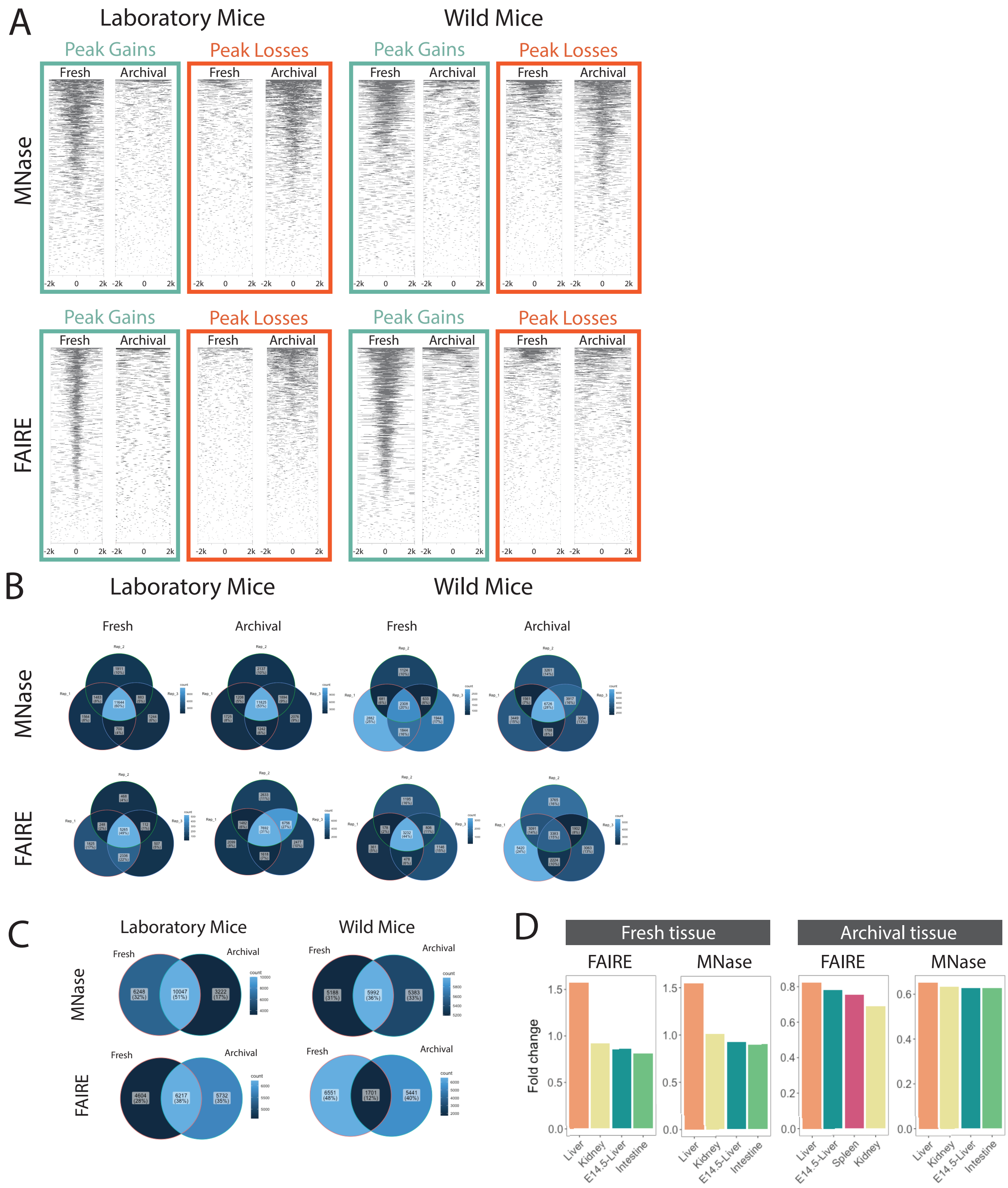

Supplementary Figure 3. Further comparisons for mouse genome-wide signal

A. Heatmap of MNase and FAIRE assay significant peak gains and losses (FDR < 0.05, log10Pval < -6) in fresh and archival tissues 2 kb either side of genome-wide transcription start sites pooled across three individuals of laboratory or wild mice. B. Venn diagrams demonstrating relative repeatability of the MNase and FAIRE assays applied to fresh and archival liver tissue among biological replicates in laboratory and wild mice. Numbers/proportions represent genes with significant peak gains for fresh tissue and losses for archival tissue (FDR < 0.05, log10Pval < -6) within 2 kb upstream of the TSS. Lighter colours indicate higher shared gene count. C. Venn diagrams showing overlap in genes identified through both fresh and archival MNase and FAIRE assays applied to three replicates in laboratory and wild mice. D. Tissue enrichment within shared gene lists of genes with pooled occupancy signal changes (Fresh = gains; Archival = losses) in the FAIRE and MNase assays from pools of three laboratory mice.

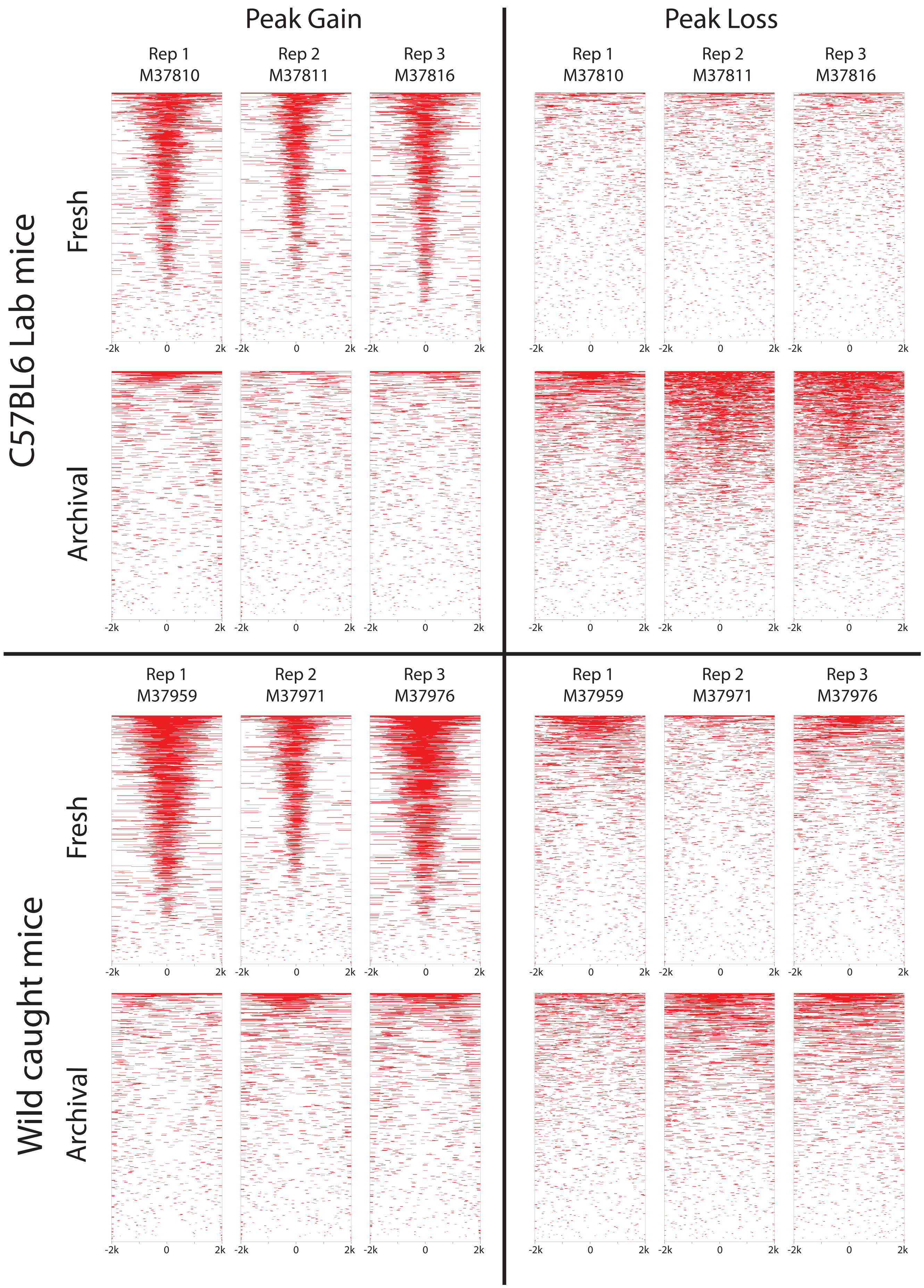

Supplementary Figure 4. Genome-wide FAIRE signal for individual mice

Heatmaps of FAIRE assay significant peak gains and losses (FDR < 0.05, log10Pval < -6) in fresh and archival tissues 2 kb either side of genome-wide transcription for three individuals of C57BL6 laboratory or wild caught mice.

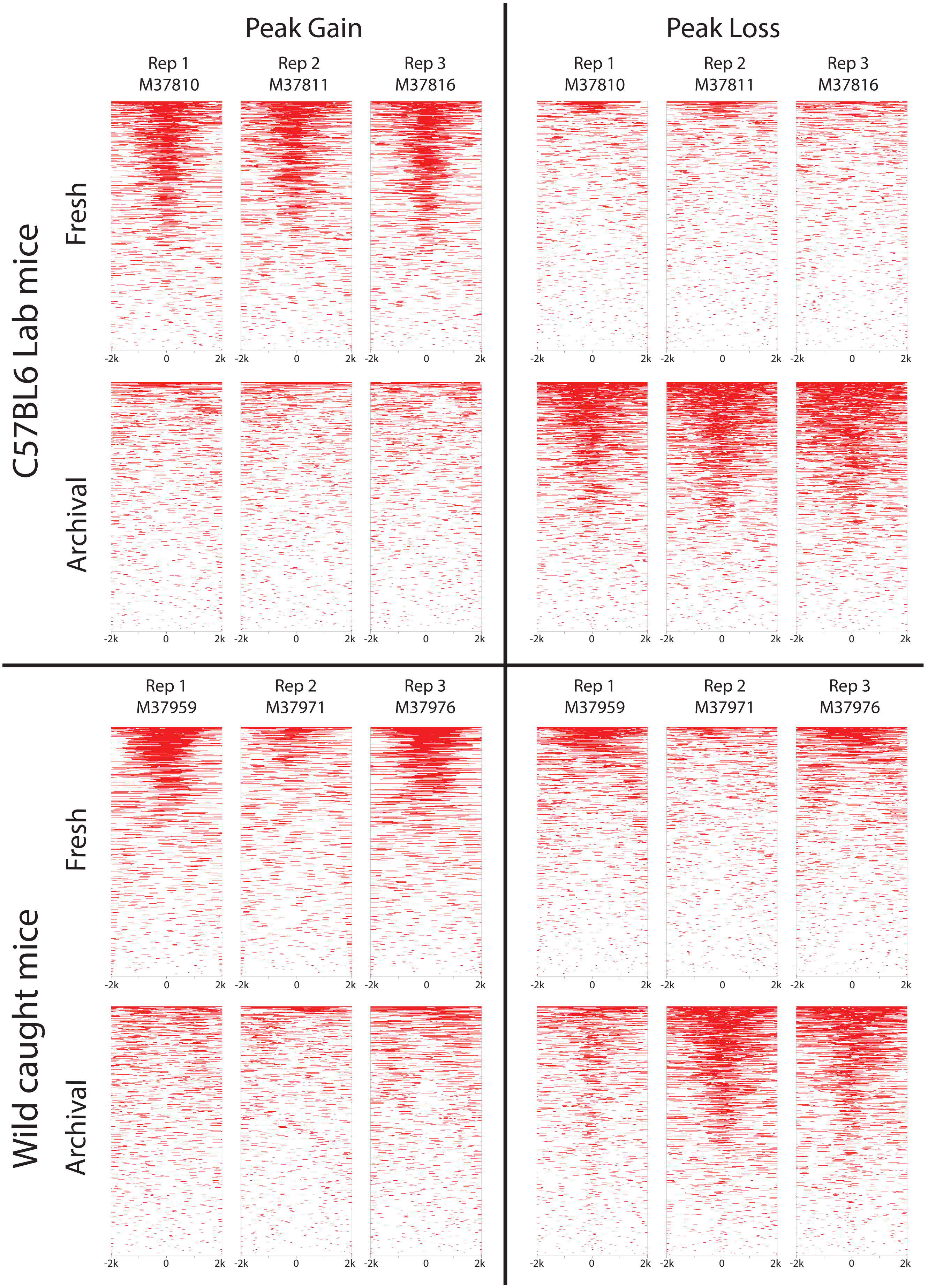

Supplementary Figure 5. Genome-wide MNase signal for individual mice

Heatmaps of MNase assay significant peak gains and losses (FDR < 0.05, log10Pval < -6) in fresh and archival tissues 2 kb either side of genome-wide transcription for three individuals of C57BL6 laboratory or wild caught mice.

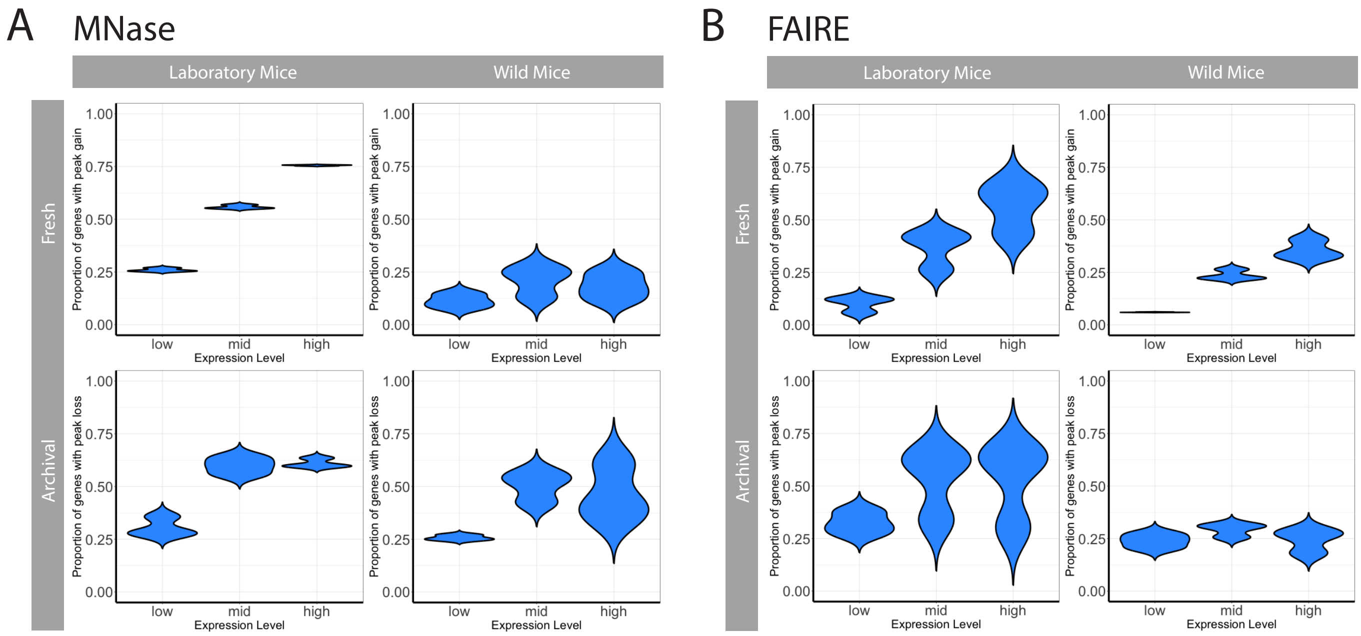

Supplementary Figure 6. Gene expression influences MNase signal detection in fresh and archival tissues

Binning genes by expression level expressed as zFPKM (low < -2; -2 > mid < 2; high > 2), we calculated the number of genes with significant (FDR < 0.05, log10Pval < -6) peak gains (fresh tissue) or losses (archival tissues) within 2 kb upstream of the TSS. Shown are violin plots for (A) MNase assay signal and (B) FAIRE from fresh and archival liver tissue among three biological replicates in laboratory and wild-caught mice. Each individual's proportions were calculated binning genes according to their individual gene expression data.

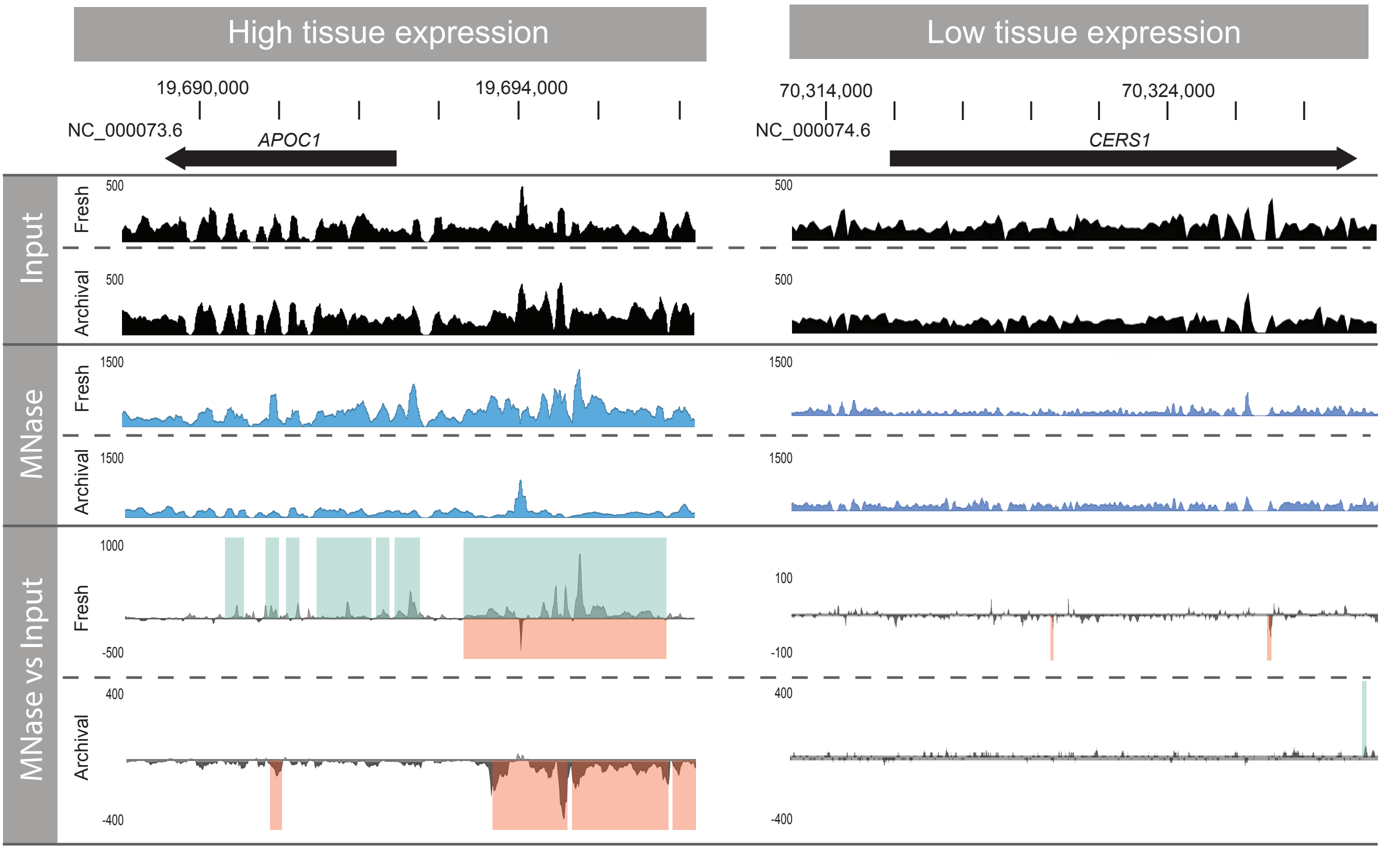

Supplementary Figure 7. Expression dependent occupancy in mouse liver

Pooled occupancy values as wiggle traces (DANPOS3 dpeak function) for input (black) and MNase (blue) as well as differential MNase signal over input control (grey) for fresh and archival Mus musculus liver tissue. Occupancy values and signal changes are shown upstream of a gene highly expressed in liver (APOC1, FPKM = 38,660) and with low expression in liver (CERS1, FPKM = 0) as measured by RNA-Seq analysis of fresh tissue. Green and orange shading upon the differential signal panel represent significant (FDR < 0.05, log10Pval < -6) peak gains or losses across three individuals detected by DANPOS3.

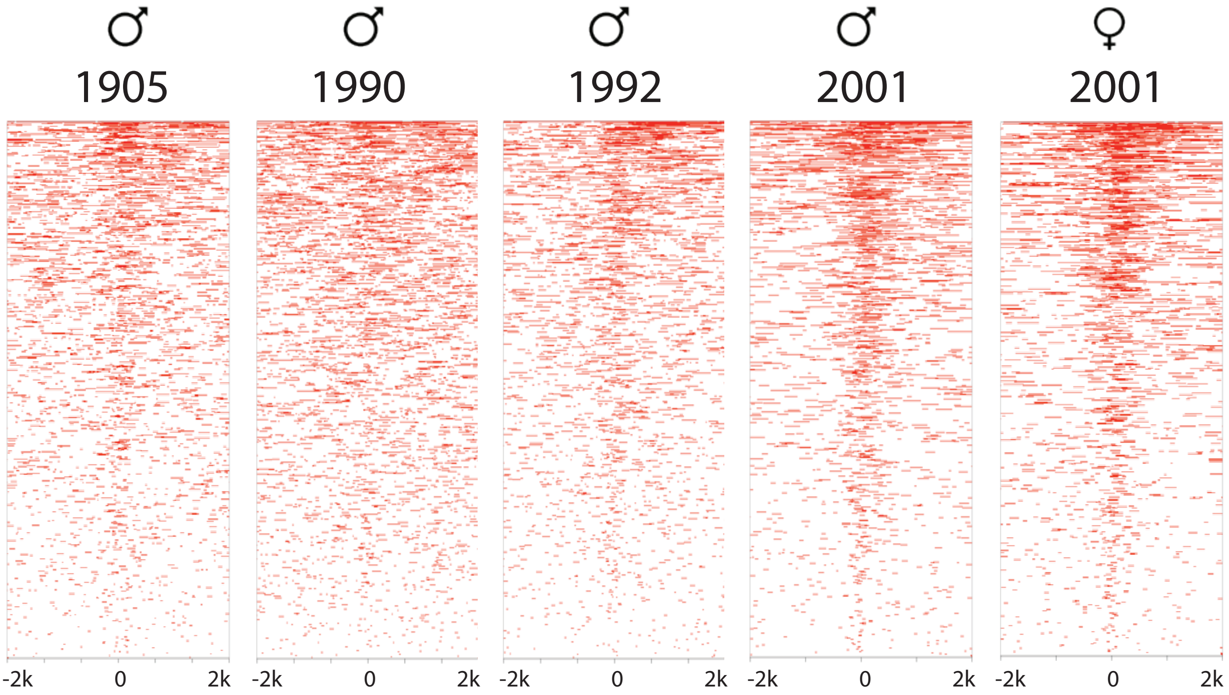

Raw reads (M)	146	151	171	154	162
% Reads mapped	40	28	32	58	49
Mean genome cover	5X	3.2X	4.3X	8.1X	7.1X

Supplementary Figure 8. Individual eastern water dragon DANPOS3 peak heatmaps

Heatmaps of MNase assay significant peak losses (FDR < 0.05, log10Pval < -6) in archival eastern water dragon liver tissues 2 kb either side of genome-wide transcription start sites (TSS). Enrichment of peak losses are observed around TSS for all individuals. Below each individual sample we have included the number of raw read pairs (millions), the percentage of raw reads mapping to the reference genome, and the mean genome coverage after de-duplication and GC correction.
