# Supplementary material for: Century-old chromatin architecture revealed in formalin-fixed vertebrates
Source: Nat Commun. 2024 Jul 29;15:6378. doi: 10.1038/s41467-024-50668-4 (PMC11286846; doi:10.1038/s41467-024-50668-4)
Supplement: Supplementary file 3 — Description of Additional Supplementary Files [file 41467_2024_50668_MOESM3_ESM.pdf]

Century-old chromatin architecture revealed in formalin-fixed vertebrates

Description of Additional Supplementary Files

Supplementary Data 1: List of all reagent sources.
