# Supplementary material for: Century-old chromatin architecture revealed in formalin-fixed vertebrates
Source: Nat Commun. 2024 Jul 29;15:6378. doi: 10.1038/s41467-024-50668-4 (PMC11286846; doi:10.1038/s41467-024-50668-4)
Supplement: Supplementary file 5 — Reporting Summary [file 41467_2024_50668_MOESM5_ESM.pdf]

Corresponding author(s): *Double-anonymous peer review submissions: write DAPR and your manuscript number here instead of author names.*

Last updated by author(s): *YYYY-MM-DD*

Reporting Summary

Nature Portfolio wishes to improve the reproducibility of the work that we publish. This form provides structure for consistency and transparency in reporting. For further information on Nature Portfolio policies, see our Editorial Policies and the Editorial Policy Checklist.

Statistics

For all statistical analyses, confirm that the following items are present in the figure legend, table legend, main text, or Methods section.

n/a	Confirmed	
☐	☒	The exact sample size (n) for each experimental group/condition, given as a discrete number and unit of measurement
☐	☒	A statement on whether measurements were taken from distinct samples or whether the same sample was measured repeatedly
☐	☒	The statistical test(s) used AND whether they are one- or two-sided<br>*Only common tests should be described solely by name; describe more complex techniques in the Methods section.*
☒	☐	A description of all covariates tested
☐	☒	A description of any assumptions or corrections, such as tests of normality and adjustment for multiple comparisons
☐	☒	A full description of the statistical parameters including central tendency (e.g. means) or other basic estimates (e.g. regression coefficient) AND variation (e.g. standard deviation) or associated estimates of uncertainty (e.g. confidence intervals)
☐	☒	For null hypothesis testing, the test statistic (e.g. F, t, r) with confidence intervals, effect sizes, degrees of freedom and P value noted<br>*Give P values as exact values whenever suitable.*
☒	☐	For Bayesian analysis, information on the choice of priors and Markov chain Monte Carlo settings
☒	☐	For hierarchical and complex designs, identification of the appropriate level for tests and full reporting of outcomes
☐	☒	Estimates of effect sizes (e.g. Cohen's d, Pearson's r), indicating how they were calculated

Our web collection on statistics for biologists contains articles on many of the points above.

Software and code

Policy information about availability of computer code

Data collection	*Provide a description of all commercial, open source and custom code used to collect the data in this study, specifying the version used OR state that no software was used.*

Data analysis	Repeat masking of reference genomes: RepeatMasker v.4.1.0<br>Computation of raw sequencing read quality metrics: FastQC v.0.11.8<br>File conversion, deduplication, insert length calculation: PICARD v2.9.2<br>UMI processing: FGBio v.1.3.0<br>Raw read alignment: ngskit4b tool suite version 200218<br>GC bias computation and correction, summary matrices of Pearson correlations: deepTools version 3.5.1<br>Alignment visualisation: CLC Genomics Workbench v21<br>Occupancy value calculation: DANPOS3<br>Peak annotation: R packages ChIPseeker, GenomicFeatures and GenomicRanges<br>RNA-Seq alignment and FPKM calculation: Tuxedo pipeline<br>FPKM to zFPKM transformation: R package zFPKM<br>Differential expression analysis: Trinity edgeR pipeline<br>Venn Diagrams: R package ggVennDiagram<br>GO enrichment in yeast: EnrichR<br>Tissue specific enrichment in mouse: TissueEnrich<br>TSS heatmaps: R package ChIP seeker<br>PCA: base R prcomp function

For manuscripts utilizing custom algorithms or software that are central to the research but not yet described in published literature, software must be made available to editors and reviewers. We strongly encourage code deposition in a community repository (e.g. GitHub). See the Nature Portfolio guidelines for submitting code & software for further information.

Data

Policy information about availability of data

All manuscripts must include a data availability statement. This statement should provide the following information, where applicable:

- Accession codes, unique identifiers, or web links for publicly available datasets
- A description of any restrictions on data availability
- For clinical datasets or third party data, please ensure that the statement adheres to our policy

The raw sequencing data generated and analysed in this study are archived in the CSIRO Data Access Portal (https://data.csiro.au/) in collections 54007 (mouse data), 51669 (yeast) and 59757 (eastern water dragon). Both the raw sequencing data and processed data (.wig) files are available from the Gene Expression Omnibus database under accession numbers GSE256156 (yeast FAIRE and MNase), GSE256160 (yeast RNA-Seq), GSE261169 (mouse FAIRE and MNase), GSE256158 (mouse RNA-Seq), and GSE256157 (water dragon).

Links to the collections on the CSIRO DAP:
Yeast - https://doi.org/10.25919/ckzk-nr22
Mouse - https://doi.org/10.25919/syr8-gb79
Water dragon - https://doi.org/10.25919/eezr-h546

Research involving human participants, their data, or biological material

Policy information about studies with human participants or human data. See also policy information about sex, gender (identity/presentation), and sexual orientation and race, ethnicity and racism.

Reporting on sex and gender	N/A
Reporting on race, ethnicity, or other socially relevant groupings	N/A
Population characteristics	N.A
Recruitment	N/A
Ethics oversight	N/A

Note that full information on the approval of the study protocol must also be provided in the manuscript.

Field-specific reporting

Please select the one below that is the best fit for your research. If you are not sure, read the appropriate sections before making your selection.

☒ Life sciences ☐ Behavioural & social sciences ☐ Ecological, evolutionary & environmental sciences

For a reference copy of the document with all sections, see nature.com/documents/nr-reporting-summary-flat.pdf

Life sciences study design

All studies must disclose on these points even when the disclosure is negative.

Sample size	For this study, we did not have previous experimental data to conduct a power analysis for determining our needed number of replicates. We selected a sample size of 3 for both the yeast and mouse experiments as a starting point.
Data exclusions	We conducted routine removal of PCR and optical duplicates from the sequencing alignments.
Replication	We have not yet verified the results through replication of the study design.
Randomization	With the laboratory mice, we selected three individuals at random from a stock of sex and age-matched preserved specimens. With the wild mice, which varied in size more so than the laboratory mice, we selected three larger specimens which had been collected from the wild within the same 3 week period. For the water dragons, we used tissues from 5 available specimens.
Blinding	Blinding was not implemented in this study design.

Reporting for specific materials, systems and methods

We require information from authors about some types of materials, experimental systems and methods used in many studies. Here, indicate whether each material, system or method listed is relevant to your study. If you are not sure if a list item applies to your research, read the appropriate section before selecting a response.

Materials & experimental systems

n/a	Involved in the study	
☒	☐	Antibodies
☐	☒	Eukaryotic cell lines
☒	☐	Palaeontology and archaeology
☐	☒	Animals and other organisms
☒	☐	Clinical data
☒	☐	Dual use research of concern
☒	☐	Plants

Methods

n/a	Involved in the study	
☒	☐	ChIP-seq
☒	☐	Flow cytometry
☒	☐	MRI-based neuroimaging

Eukaryotic cell lines

Policy information about cell lines and Sex and Gender in Research

Cell line source(s)	We used Saccharomyces cerevisiae strain BJ5464 auxotroph ΔURA3
Authentication	Cell lines were not authenticated as the experiments did not rely on functions specific to the cell line
Mycoplasma contamination	Cell lines were not tested for mycoplasma contamination as the experiments did not rely on functions specific to the cell line
Commonly misidentified lines (See ICLAC register)	N/A

Animals and other research organisms

Policy information about studies involving animals; ARRIVE guidelines recommended for reporting animal research, and Sex and Gender in Research

Laboratory animals	Male Mus musculus, strain C57BL/6, aged 17-18 weeks, sourced from Australian BioResources and sacrificed upon arrival
Wild animals	Wild Mus musculus specimens were donated to our study by the CSIRO Health and Biosecurity Rodent Management Team. The mice had been live caught using Longsworth single-capture mouse traps. Healthy animals were transported via air-conditioned vehicle within the traps and supplied bedding, wheat and apple. Mice were then individually housed in clear cages at ambient temperature, humidity and natural day/light cycle and provided with food, water and bedding material. Mice were used for a maximum of 4 months in non-invasive behavioural tests. Due to restrictions on releasing exotic species, the animals were humanely sacrificed via cervical dislocation and preserved for archiving in the ANWC. We dissected frozen liver tissue from three eastern water dragons euthanised due to injury in accordance with Queensland Department of Environment and Sciences permit WA0038029.
Reporting on sex	This study does not specifically look at the effect of sex, however, we do detect a sex effect which highlights the influence of sex on gene activity and underscores the importance of sex-matching in future studies. For the mice, sex was confirmed visually prior to dissection. For the water dragons, sex was determined from the specimen metadata.

Field-collected samples	N/A
Ethics oversight	Ethics approval was received for our work with laboratory (protocol number number 2017-34 - CSIRO) and wild (protocol number 2018-46 - CSIRO) mice as well as eastern water dragon (protocol number ANA20161 - University of Sunshine Coast) through the Australian Ethics Committee.

Note that full information on the approval of the study protocol must also be provided in the manuscript.

Plants

Seed stocks	N/A
Novel plant genotypes	N/A
Authentication	N/A
